# Trajectories of adolescents admitted to the emergency department for suicidal behavior between 2019 and 2021: impact of the Covid-19 pandemic, descriptive analysis and predictive model development

**DOI:** 10.1192/j.eurpsy.2025.1111

**Published:** 2025-08-26

**Authors:** A. Bounan, M. Cayla, H. Kerbage, N. Franc, E. Jeziorski, D. Purper-Ouakil

**Affiliations:** 1 Université de Montpellier, Montpellier, France

## Abstract

**Introduction:**

Adolescent suicidal behaviors have seen a marked increase in incidence, particularly following the onset of the Covid-19 pandemic (Revet *et al.* Eur Child Adolesc Psychiatry 2023; 32 249–256). This surge has presented challenges for emergency and psychiatric services. It highlighted the need for improved understanding of predictive and protective factors linked to recurrent suicide attempts.

**Objectives:**

This study aims to analyze the clinical trajectories of adolescents admitted to emergency departments for suicide attempts between 2019 and 2021 and to identify predictors of recurrence, with a particular focus on conditions that may elevate the risk of repeat attempts (Tomaszek *et al.* Front Psychiatry 2024).

**Methods:**

We conducted a retrospective cohort study at the Montpellier University Hospital, examining pediatric emergency visits related to suicide attempts over three consecutive years (2019–2021). The dataset included patient demographics, psychiatric diagnoses, treatments prescribed, and hospitalization metrics such as duration and readmission frequency. Statistical analyses employed a multivariate linear regression to identify significant predictors of recurrence.

**Results:**

The incidence of emergency visits for adolescent suicide attempts rose elevenfold from 2019 to 2021, reflecting the global trends in mental health deterioration post-Covid-19 (Lespes-Hislen *et al.* 2023). Recurrence rates were notably higher among patients initially admitted in 2021, with 54% of these adolescents re-presenting for subsequent suicide attempts, indicating a persistent crisis in mental health among this demographic. The selected model identified ADHD diagnosis, the use of mood stabilizers, and prolonged hospitalization as significant predictors of recurrence. In the regression model, each additional day of hospitalization was associated with an estimated increase in recurrence risk of 0.16 additional attempts per 1-day of hospital stay.

**Conclusions:**

This study confirms an increase in suicidal behaviors after the pandemic and highlights the importance of personalized care, especially for adolescents with ADHD. The association between hospitalization duration and recurrence raises questions about the effectiveness of prolonged hospital stays in this population.

**Disclosure of Interest:**

None Declared

